# Mechanistic decomposition and reduction in complex, context-sensitive systems

**DOI:** 10.3389/fpsyg.2022.992347

**Published:** 2022-11-07

**Authors:** Daniel C. Burnston

**Affiliations:** Philosophy Department, Tulane University, Tulane Brain Institute, New Orleans, LA, United States

**Keywords:** mechanism, emergence, context-sensitivity, network organization, complex systems

## Abstract

Standard arguments in philosophy of science infer from the complexity of biological and neural systems to the presence of emergence and failure of mechanistic/reductionist explanation for those systems. I argue against this kind of argument, specifically focusing on the notion of context-sensitivity. Context-sensitivity is standardly taken to be incompatible with reductionistic explanation, because it shows that larger-scale factors influence the functioning of lower-level parts. I argue that this argument can be overcome if there are mechanisms underlying those context-specific reorganizations. I argue that such mechanisms are frequently discovered in neuroscience.

## Introduction

Biological systems are complex. They are multi-scale, heavily interactive, and context dependent. In this paper, I will assess the ramifications of these facts for reductive and mechanistic explanation. One common reaction to the recognition of complexity is to deny that mechanistic and reductive explanations are possible, or, more weakly, to suggest that their scope is extremely limited. Instead, it is often argued, we should embrace an emergence thesis, and concomitantly a commitment to using distinct forms of explanation for emergent properties in complex systems.

I will question this line of thinking. In particular, I will question the idea that widespread context sensitivity across scales is tantamount to emergence. I will focus on the brain. Neural systems have recently been recognized to involve complex interactions between their parts, multi-functionality of individual parts, and context sensitive forms of organization (Anderson, 2014; Burnston, 2016a,b, 2021; de Wit and Matheson, 2022). As such, the brain, and the cognitive phenomena to which it gives rise, provide a good test case for assessing emergentist claims.

I will endorse, with others in the literature (Silberstein, 2021), the idea that functional decomposition and localization are the *sine qua non* of mechanistic explanation. The question is then best phrased as: do widespread context sensitivity and multi-scale relations in neural systems require us to embrace emergence and abandon localization and decomposition as explanatory strategies? I will argue that they do not, so long as mechanisms by which context is recognized and used to implement functional reorganization are discoverable. If so, then the system is mechanistically explicable despite context sensitivity.

I begin (section 2) by laying out some of the intersecting dialectical dimensions that comprise the current debate. I endorse a pragmatic construal of the debate along the lines above, and offer a version of reductionism that is based on what I call the pragmatic downward pull of research – the idea that it is normatively better to seek and discover mechanisms at lower levels that comprise one’s phenomenon of interest. In section 3 I argue, using some toy examples, that there is nothing *inherently* emergentist about context-sensitivity and multi-scale structure. I then go on (section 4) to illustrate a variety of mechanisms for context-recognition and functional reorganization in the brain, and I suggest that seeking these mechanisms is required for understanding how the brain produces cognitive phenomena. I then (section 5) give my general interpretation of the cases and consider some possible objections. Section 6 concludes.

## Dimensions of the reduction and emergence debate

Something of an anti-reductionist consensus has arisen in philosophy of biology (Huttemann and Love, 2011; Kaiser, 2015; Brigandt et al., 2018). There are many reasons for this. For one, many have recognized that traditional reductionist approaches fare badly in accounting for the multi-scale organization involved in biological systems (Wimsatt, 2006). Another is the increased importance, within the last few decades, of dynamical systems and network-based approaches in understanding, e.g., genetic and neural systems (Green et al., 2018; Huneman, 2018). These approaches paint biological systems as inherently interactive and multi-scale, and as falling into classes of topological organization.

The best response a reductionist can make in these circumstances, in my view, is to admit that traditional reductive forms of explanation are indeed hopeless in light of these developments, but to suggest that traditional forms are not the only possible ones. For instance, traditional reductionist approaches have tended strongly towards “atomism” (Burnston, 2021), a view on which explanation proceeds by first discovering the intrinsic functional properties of the relevant lower-level parts, and then (and only then) explaining the properties of the system as interactions between those intrinsic properties. This style of explanation has indeed characterized some eras of investigation in biology and neuroscience, but it is not obvious (and, I will argue, not true) that this is the only way a reductionist thesis might be phrased. Why not come up with more complicated reductive schemas in an attempt to account for complexity in these systems?

I will assume that reductionist and mechanistic approaches are closely allied (although there are non-reductionist accounts of mechanisms; see Couch, this issue), in the sense of “explanatory reductions” (Sarkar, 1992).^1^ This is because mechanistic approaches are committed to decomposition and localization of system properties at lower levels. The question, on this view, is whether a sophisticated enough, but still genuinely mechanistic, account can be given that integrates with dynamical and network descriptions in a productive way. In the remainder of this section, I will lay out some of the extant dialectic surrounding the issue, and then give my preferred reading of reductionism in light of that extant discussion.

### Some extant dialectical dimensions

While I make no claim to exhaustiveness, the following are some of the important dimensions surrounding debates about emergence. Note that these are related in numerous ways, and intuitions along one may correlate with intuitions along others. I do not plan to explore the details of this space in full, but instead to lay out some relevant issues so as to better express my version of the reductionism thesis in the next subsection.

#### Strong vs. weak emergence

Strong emergence is a view of emergence on which there is a discontinuity in nature between lower-level and higher-level phenomena. On traditional views, this has been expressed as the idea that new laws of nature apply to higher-level phenomena, that are not determined by basic physical laws. This has come to be viewed, with some exceptions (Boogerd et al., 2005), as too strong of a position. Views that posit weaker kinds of emergence, on the other hand, posit that there is no discontinuity in nature, but instead that certain organizational features at higher levels are emergent, even if they are ultimately the outcome of basic physical processes. These views have to be careful not to devolve into being too weak – i.e., they should not take basic aggregative and relational properties to be emergent. Take the property of being *five stones in a box*. This property is, trivially, not a property of any of the individual stones or of the box. But no reference to anything beyond the basic physical objects and their arrangement is required to account for the existence and causal powers of this property. Hence, views of emergence must situate themselves with regards to what kind of distinctions they posit between levels, and when those differences are robust enough to justify positing of emergence.

#### Ontological vs. epistemic emergence

If emergence occurs, is it a feature of the world or a feature of human descriptions of the world? On the former view, certain natural systems are organized such that novel higher-level properties are generated in those systems, and hence emergence is a feature of the world. On the latter view, emergence is an epistemic phenomenon – that is, perceived differences between levels are the result of limitations of human classification, imagination, computational resources, etc., rather than any independent feature of the world. Emergence, on this kind of position, is the outcome of epistemic limitation and/or convenience. We posit emergence when we find it convenient or necessary to move away from descriptions of a given type at a given level, to descriptions of a different type at a higher level. A view on emergence must make clear whether it is positing an ontological or epistemic version.

#### Definitional vs. pragmatic argument

This distinction is not so much a distinction between *types* of emergence, but instead a distinction between kinds of arguments given for emergence. On the definitional approach, one posits that emergence is co-extensional with complexity of certain kinds. One then attempts to define emergence in terms of the relevant kinds of complexity in natural systems. For instance, consider Deacon’s 2006 claim that one should define “a technical sense of emergence that explicitly describes a specific class of causal topologies.” On this kind of view, emergence is taken to just be the way that complexity is to be understood, and hence complexity is, definitionally, evidence for emergence. The pragmatic approach is much different.

Pragmatic arguments involve abductions over scientific practice and explanation. The necessity of different descriptions at different levels in science, pragmatic arguers suggest, is evidence that emergence is present – otherwise we would be able to close the explanatory gaps between different types of explanation at distinct levels. Note that a pragmatist need not be an epistemicist (although they may be). It is perfectly compatible with pragmatism to suggest that the best explanation for the presence and necessity of distinct modeling practices in the sciences is that emergence occurs in the world.

#### Emergence vs. mechanism

Is emergence incompatible with mechanistic and reductive explanation? Most views suggest that there is at least a strong tension between these positions. But this is not obviously the case. Bechtel (2016) has asserted that “reductionists must be holists too!,” arguing that any worthwhile explanation of a system at a lower level must make reference to systemic properties and organization – otherwise, one would never know *which* kinds of organization must be implemented at the lower level. Moreover, it has always been a part of Bechtel’s program that mechanistic explanations must go hand-in-hand with dynamical explanations in order to account for phenomena (Bechtel and Abrahamsen, 2010). And he has recently applied this further to network explanation (Bechtel, 2019). Similarly, mechanists such as Kaplan and Craver (2011) have argued that dynamical models, to attain explanatory status, must be “mapped” to mechanistic descriptions.

Other recent mechanist proposals have embraced the ideas that some take to hallmarks of emergentist positions. For instance, in previous work I have argued extensively that functional decomposition and localization should themselves be contextualized to behavioral and physiological circumstances (Burnston, 2016a, 2021). On this position, there is no tension between context-sensitivity and mechanistic/reductive explanation (*cf.* Delehanty, 2005; for further discussion, see Gillett, 2016). Levy and Bechtel (2016) have suggested that mechanism existence and identity can shift over time – mechanisms may pop into and out of existence, change their organizational properties, etc. The main danger with this dimension is that the dispute risks dissolving into a semantic one, with mechanists and emergentists both recognizing all of the same facts and simply employing different verbiage to describe them (Silberstein, 2022).

### My construal of the debate

My construal of the debate begins by focusing on the *definitional* vs*. pragmatic* dimension. In my view, the only productive version of the debate is one that takes pragmatics as its starting point. If the question of emergence is definitional, then there simply *is no debate to be had*. If emergence is co-extensional with complexity, then the presence of complexity entails the presence of emergence. We must either (i) accept that mechanism/reductionism is false full stop, or (ii) redefine mechanism and reductionism to be compatible with emergence. There is no possibility of reconstruing mechanistic/reductionist positions along the lines just discussed, so as to both be compatible with complexity and to be an alternative to emergentist views. Basically, anyone who recognizes complexity in biological systems is an emergentist of some type, anyone who does not recognize it is naïve, and we can all go to the pub.

As fun as the pub sounds, this is not a very productive way to have a philosophical dispute. Hence, the pragmatic phrasing of the debate is the way to go. On this construal, we have all sorts of interesting things to consider, including scientific practice and explanatory frameworks, and these can serve as genuine evidence for theses about emergence and reductionism. Pragmatism also leaves open a lot of room for how one construes the other dimensions. As noted, pragmatic arguments are abductions from scientific practice and explanation, and emergence is affirmed (or denied) as the best explanation for the nature of those practices. This is compatible with having stronger or weaker views of the kind of emergence one must posit to explain those practices, and with whether one thinks that explanation posits ontic or purely epistemic emergence. Importantly, it also gives a way of overcoming the worry that differences between mechanistic and emergentist views are purely semantic. Since the pragmatic approach is based on abduction from certain forms of scientific practice, mechanistic and emergentist views should give genuinely distinct descriptive and normative readings of scientific investigation.

Given my construal of the debate, I will take as my stalking horse throughout this paper a recent view of emergence developed by Silberstein et al. (Bishop et al., forthcoming). On this view, called “contextual emergence,” widespread context-sensitivity of systems, and the explanations that scientists resort to in order to explain properties of these systems, provide support for an emergentist thesis. Silberstein et al., quite rightly note that context-sensitivity is widespread in biological and physical systems. They describe emergence as necessary to account for the *multi-scale constraints* and the *topological structure* of these systems.

Multi-scale constraints are instances in which organizational properties at higher-levels influence or determine the properties of lower-level entities. Topological structure means that whole systems implement global structures that are characterizable independently of the lower-level components that comprise them – usually, these kinds of explanations make use of the resources of graph theory, and the types of topologies it describes. These can include organizations such as being a *small-world* network, or exhibiting a *rich club* organization (discussed further below), each of which are present in many different kinds of systems with vastly different component parts. In a context-sensitive system, Silberstein et al., argue, topological properties and multi-scale constraints determine how a system can behave in new contexts. As such, “Contextual constraints represent both the screening off and opening up of new areas of modal space, i.e., degrees of freedom, and thereby new patterns” (Silberstein, 2022).

I target this view because it is the first view of emergence that I am aware of that makes context-sensitivity one of its main tenets and sources of argument (although see Huttemann and Love, 2011). Since I agree about the context-sensitivity of biological organization, this is a productive starting point. Moreover, the authors are admirably clear about their position on the dialectical dimensions just discussed. First, like me, they propose to make pragmatics the main argumentative strategy. Their primary argument is that the nature of science shows the context-sensitive, multi-scale, and topological nature of the systems under study. Hence, I agree with them on the way the arguments should proceed.

Silberstein et al., characterize contextual emergence as *moderately strong*, *both ontological and epistemic*, and as *in conflict with mechanistic/reductive analysis*. They are moderately strong in that they think genuine new forms of organization emerge at the global/topological level, and interact with lower-level processes, particularly by constraining them. This is not normal strong emergence in that it posits no breaks in nature, no fundamentally new laws, etc., and it is not inexplicable – there is simply a new type of fact when systems are arranged so as to implement context-sensitivity, multi-level constraints, and topological organization.

But the view is also not among the weakest in that it does not simply posit that any relational or aggregative processes are emergent. The constraints exerted on gas particles by the wall of a container, for instance, are not emergent on their view. In contrast, Silberstein et al., offer the example of Rayleigh–Bénard convection, in which fluid particles subject to a temperature gradient within a container form subsisting units that move in regular patterns. On this view, it is the context of the container and the temperature gradient which produces a higher-level organization, which then constrains lower-level behavior, canceling out perturbations in individual particles to retain the higher-level structure.

Similarly, while the view is pragmatic, it is not purely epistemic. Silberstein et al. think it is a fact *about nature* that systems are organized in the way they propose, and that this is the best explanation for the multi-scale and topological explanations scientists give. As such, they are against any purely epistemic view that posits emergent properties as the result of explanatory convenience. Topological properties are not, for instance, merely abstractions over lower-level organizations, but are themselves a distinct type of property that systems can instantiate. Lastly, they take contextual emergence to be in conflict with mechanistic explanation, specifically because they think decomposition and localization fail for such systems. They thus suggest a typology of explanations. Multi-scale topological explanations, on this view, are *distinct from* and *explanatorily independent* of mechanistic ones. In particular, if one adopts a topological style of explanation, one eschews decomposition and localization, and vice versa.

Neural systems are among the explanatory targets of contextual emergence. Following on the earlier work of Chemero and Silberstein (2008) and Silberstein and Chemero (2013). Silberstein et al., posit that neural systems meet the classification of contextual emergence, and therefore that mechanistic analysis is either incorrect when applied to these systems or not fruitful. In support of the contextual emergence thesis with regards to neuroscience, Bishop et al. (forthcoming) list a wide range of facts about the multi-scale nature of the brain, including neural modulation at the cell level, neural synchrony at the circuit level, and the dependence of development on social context as evidence in favor of contextual emergence. Silberstein (2021) further discusses the widespread plasticity of neural systems (*cf.* Zerilli, 2020). In other work, Silberstein and Chemero (2013) and Silberstein (2021)suggest that cognitive phenomena, including those interrupted in psychiatric conditions, are dependent on network organization, and therefore not explicable in terms of localization and decomposition.

Silberstein (2021) has claimed that the attempts of mechanists embrace complexity rob mechanism of any distinctive content. That is, one can only make mechanism compatible with complexity by so weakening decomposition and localization (as well as the conditions on mechanism identity) that they are simply redescribing contextual emergence in mechanist language, hence rendering the debate verbal. So, in order to adjudicate the debate, we need a characterization of mechanistic/reductive explanation that would resist having purely semantic differences with contextual emergence. And we would need to know what kind of evidence to look for in scientific practice and explanation to determine whether that characterization is met. I propose the following.

I characterize reductionist/mechanistic explanation according to what I call *pragmatic downward pull*. That is, reductive explanation is the normative principle that it is better to understand the lower-level mechanistic organization in one’s system of interest, even in the kinds of systems emergentists cite, and that it is not possible to explain phenomena entirely without doing so. We can now use this characterization to re-phrase the debate between the mechanist/reductionist and the contextual emergentist. The question is, are circumstances in which context affects the organization of a system, in which network organization is relevant, etc., inherently circumstances in which mechanistic and reductive frameworks of explanation are either *not possible* or *not desirable*?

It is worth pausing to note the ways in which this formulation of reductionism differs from traditional approaches. This approach is neither atomistic nor an instance of “nothing but-ism.” That is, it does not suggest that explanation can only rely on intrinsic properties of lower level parts; nor does it suggest that we must know all of the relevant lower-level information before we individuate system-level properties; nor does it deny that the resources of, e.g., topological or dynamical models can contribute explanatorily important, distinct information. What it does require, however, is that these system-level properties and models still need to be understood in terms of localization and decomposition. Within a topological organization, for instance, we need to understand how distinct components within that system contribute differentially to the phenomenon of interest; one must link the multiple kinds of explanation, and “connect” functional distinctions within the system to the phenomenon of interest by linking together the causal path that produces the phenomenon (Bickle and Kostko, 2018). So, reductionism construed as pragmatic downward pull offers a substantive view that is genuinely distinct from emergentist ones.

I will only focus on neuroscientific explanation here. In keeping with the pragmatist approach, the success of a position in the debate depends on whether it provides the right descriptive and normative view of how the best neuroscience works. In what follows, I argue that my construal of pragmatic downward pull is the best description of investigation into context-sensitive and network-mediated neural systems.

In particular, I will suggest that scientists seek a particular kind of mechanism when analyzing such systems – that is, they investigate *mechanisms that recognize context and implement new forms of organization*. If these mechanisms can be found, I argue, then we can understand shifts in context in a fully mechanistic way, and indeed we need to investigate these mechanisms in order to understand how the system works. That is, pragmatic downward pull obtains.

In section 4, I will discuss a number of context-recognition and reorganization mechanisms that neuroscientists have uncovered. Before doing so, however, I want to set the stage a bit by considering some toy examples.

## Context, topology, and constraints – Inherently emergent?

This section will be an exercise in deck stacking – or, at least, deck evening. I want to imagine some simple toy systems and ask whether, first, they can exhibit the properties that interest emergentists, and second whether they must be construed as implementing emergence.

One of the longest running daytime TV shows in the US is *The Price is Right*. As part of the show, contestants participate in carnival-style games, one of which is (or at least used to be) *Plinko*. In a *Plinko* system, one drops a ball from the top of a board, and the ball falls through a series of obstacles, ending up in one of several boxes at the bottom, each box representing a prize. The obstacles on the board are set in a lattice organization, so that the movement of the ball is a kind of random walk through the obstacles.

Here, obviously, the lattice affects the movements of the ball. But each of the interactions of the ball with individual obstacles is perfectly well-explained by basic causal interactions between them. The ball exhibits a kind of path dependence. The nature of its interaction with the first obstacle positions it so that it then has a certain interaction with the next obstacle, which positions it for the following one, etc. I submit that if there is emergence in the *Plinko* system, it is only of the weakest kind, where the arrangement of the obstacles shapes the directions in which the ball can go, but every interaction of the ball with the individual obstacles, and each *particular* path of the ball through the system, is fully explainable in terms of local interactions.

Let us imagine some slight variations to the *Plinko* framework. First, there’s no reason why gravity can be the only force moving the ball, or downward the only direction. We can imagine a multi-directional Plinko board, where fans or vacuums or whatever propel the ball from any side to any other. Second, we do not have to think of the board as constant.

Suppose that, behind the scenes, there is a lever. When someone pulls the lever, a series of gears turn the obstacles so that they are now in a new arrangement. When the lever is thrown, the obstacles move in the following way. First, they closely align into rows, creating corridors through which the ball can quickly move. However, these corridors are frequently punctuated by “clearings,” around which the ball must bounce before finding a new corridor to enter. Further, suppose that some “clearings” are only connected by corridors to a couple of other clearings, but that some are connected to many clearings. In this system, the clearings and corridors roughly mirror the nodes and edges of a network. Clearings that connect to many other clearings will be “hubs” in this network. We can further imagine a distribution of clearings such that most clearings are low in connections or “degree,” and only connected to nodes close to them, but the hub nodes are heavily interconnected with long range connections. This would be an analogue of a “small world” network. We can even imagine that the hubs are densely connected to *each other*, emulating what is called a “rich club” structure.

Once the lever is thrown, these topological facts will become relevant for the kinds of paths the ball can take. A ball in a rich club system, for instance, will likely move through the board faster, because the motive force will move it down a corridor, soon reaching a hub. Since hubs are richly connected in a rich club system, the ball will more quickly move through the board by hopping from hub to hub. Further, different *specific arrangements* – for instance, distinct spatial groupings of hubs – could each implement a rich club network. Imagine a rich club board, but one where the hub nodes are spatially clustered on one side of the board. Here, not only will ball traversals be slower than on a more spatially diffuse rich club (since balls run the risk of getting “trapped” in the rich club at one end of the board), but which *side* the ball starts on will matter. A ball placed in the rich club side will be more likely to find its way quickly to the other side of the board, due to the long range connections of the hubs, than a ball placed on the other side, that will risk wandering significantly before finding the “highway” corridors connecting hubs.

Here we have a situation where topology and context matter deeply for the “modal possibilities” of ball trajectories. A ball in the rich club board will have a much different distribution of possible trajectories. Still, I submit, there is nothing more than weakly emergent about this system. First, the changes of configuration are fully explained by the lever and gear system. This in turn modulates the way that the ball can move in new contexts (e.g., its direction of travel). But each particular trajectory is just a series of basic of basic mechanical interactions between the ball and the assorted obstacles.

What about the “topological facts” I alluded to earlier, and the fact that they are multiply realizable by distinct spatial layouts of particular boards (not to mentions by wooden versus metal obstacles, etc.)? Given the setup of the case, this cannot be sufficient motivation for positing contextual emergence. Of course, different mechanisms can be similar in many respects. Citing a similarity between them is just citing an abstract feature that they share – and, as noted above, contextual emergentists insist that features exhibiting contextual emergence are not best described as useful abstractions of mechanistic properties. Moreover, note that similarities are important until they are not – the fact that the last two boards discussed both implement rich club networks does not mean that they are the same in all relevant respects – in some contexts, the differences between their spatial distributions do matter, for instance depending on the starting point of the ball.

An important aspect of this case is that, when context changes, one can explain that change in context *via a mechanism of contextual reorganization*. In the *Plinko* system, the lever and gear system *explains* the new form of organization, and the paths of the ball through the board are then the result of interactions within that organization.^2^ These are the two properties which I think are important for assessing the debate in neural systems. If we can explain *both* how contextual changes are implemented mechanistically in a system, *and* can show functional localization and decomposition *within* a context, then mechanistic/reductive explanation is possible despite context-sensitivity. I will argue that both facts obtain in the neural case.

I am not, of course, suggesting that the *Plinko* system is straightforwardly analogous to any biological or neural system. Biological systems have much more complex forms of organization and interaction. For one, they implement connections over a distance (e.g., through signaling) rather than *via* direct physical connection. For another, they often have bi-directional or reciprocal functional connections, wherein two parts influence each other mutually. Further, biological components often respond to *ensemble* properties, such as chemical gradients or, in the neural case, background electrical potentials. But none of these facts *themselves* require that decomposition and localization must fail. It would take an extra argument that localization and decomposition are not possible in these cases.

In the next section, I suggest four different types of mechanisms that neural systems implement to manage contextual change (I’m sure there are more). The emergentist is forced into the awkward position of claiming that we *should not care* about these kinds of mechanisms – i.e., they do not contribute productively to explanation. This, I claim, is wrong.

## Context-recognition and implementation mechanisms in the brain

### Context re-mapping and invariance mechanisms

The first set of mechanisms that I will consider involve how populations of cells either *re-map* their selectivity in particular contexts, or, just importantly, how they can come to generalize or achieve invariance *within* a type of context. These kinds of mechanisms show that learning and plasticity can *implement* specific forms of functional localization within particular units in the brain, which are themselves sensitive to the context.

The first example of re-mapping comes from physiological study of hippocampal neurons in monkeys. It is well-established that hippocampal cells exhibit *mixed-selectivity*, which means that they are selective for multiple parameters of a task context or stimulus (Rigotti et al., 2013). This is true even for place cells, whose responses are dependent primarily on the organism’s spatial position. It is also the case that hippocampal cells are *variant* in their responses. This means that their responses can vary depending on the kind of environment that the organism is in, or its position in that environment. Some cells that show place-selectivity for one environment, for instance, will lose it or show a different selectivity in a different environment (Maren et al., 2013). Within a given environment, cells exhibit *phase precession*, which means that they sync to different phases of the theta rhythm in the local field potential depending on the organism’s position in the environment.

Baraduc et al. (2019), in a study in *Nature*, explored how hippocampal cells of this type could learn to generalize across superficial changes to behavioral context, where the primarily important structure of that environment stayed the same. To do this, they had monkeys explore a virtual reality maze while recording from hippocampal neurons. They first had the monkeys learn a maze where rewards were “hidden” in different locations, and a primary cue for their locations was the relative spatial position of certain landmarks. So, if (for instance) a tree was to the right of a star, the reward would be in between the two landmarks.

The key manipulation of this study was when the experimenters changed the maze, while keeping the relational positional structure the same. So, for example, rather than a tree being to the left of a star, with the reward to the right of the tree, the tree and the star could be replaced by a triangle and a square, respectively, with the reward to the right of the triangle. Further they “rotated” the maze, such that the starting point varied from the monkey’s starting point in the original maze. Intriguingly, once monkeys began to explore these kinds of mazes, versus totally novel mazes, they quickly realized that they had the same structure as the previous maze. This was shown by their rapidly learning the new maze.

Furthermore, *some* cells in the hippocampus exhibited similar selectivity properties in the structurally similar mazes after learning. In particular, these cells were selective to the current position of the monkey in the abstract structure, and its action-possibilities – e.g., re-orienting in a new direction to face the reward. Other cells in the hippocampus did exhibit re-mappings with the novel mazes, even those that shared the same abstract structure. So, the hippocampus exhibits multiple populations with selectivity properties that re-map to new contexts, but also form invariances to higher-order elements of context (e.g., spatial relations) as other aspects change.

A second example of re-mapping of this type involves not the selectivity properties of cells, but instead the structure of the population, i.e., how the population forms functional groups that are appropriate to the context. Cohen and Newsome (2008) performed a study where a sensory stimulus was of the same type across contexts, but what kind of decision a monkey had to make varied depending on the context. The stimuli were dot-motion stimuli, in which the monkey is shown a pattern of dots moving in different directions. The level of “predominant” motion can be varied depending on the correlation between dots. So, more dots moving together to the left will result in predominant motion to the left, and so on for the other directions. Neurons from area MT, an extrastriate visual area dedicated (partially; see Burnston, 2016a) to motion, were measured while monkeys viewed these stimuli and made decisions about the direction of predominant motion.

The context manipulation involved implementing distinct two-alternative forced choice tasks. One task type involved asking the monkey whether *left* or *right* had more predominant motion. Another involved asking whether *up* or *down* did. This allowed for contrasts in context to be measured within cell populations in MT, based on how they related to the choice situation. Imagine two cells, one with selectivity for “motion upward to the left,” and one with selectivity for “motion upward to the right.” If the decision that needs to be made is *up or down*, then these cells will be cooperating in the decision – each will indicate *up*. However, if they decision is between *left and right*, they will be competing – one will indicate *left* and one will indicate *right*.

The idea of the researchers was that the cell population could be differentially recruited to implement these co-operations and competitions in the right setting. In particular, they measured “noise correlation,” which is a comparison of the variance between two cells in similar trials. The reasoning here is that if two cells are part of a cooperating circuit, they will tend to vary together even in their noise properties. Intriguingly, they showed just this pattern. Two neurons of the type described above would show *increased* noise correlation in the *up or down* decision, and *decreased* noise correlation in the *left or right* decision. The authors suggest that (i) this is evidence of the neurons being in cooperative versus competitive circuits in the distinct contexts, and (ii) that the population reorganization may be due to attentional signals from more frontal areas of the cortex, shifting the population between attentional patterns for the different contexts.

In both of these cases, the populations in question exhibit plasticity and context-sensitivity. That is, they show particular selectivity or correlational variance that is sensitive to context. Of course, this is not the whole explanation, since there is still a question of how information *about* the context is relayed to the relevant populations. This brings us to the kind of mechanism discussed in the next subsection.

### Context recognition and signaling

In this subsection, I discuss examples in which a system can be decomposed into a part that recognizes the context, compared to parts that provide it input, or which it causally affects downstream.

One example is shown in fMRI studies of humans, specifically with regards to fear conditioning. Context is very important for fear conditioning, since Pavlovian conditioning can be indexed to contexts, for instance when a mouse exhibits freezing in a cage where it has previously experienced a foot shock. The role of the hippocampus in context-based fear conditioning is well established physiologically in animal studies. Maren et al. (2013) cite a range of studies in which aversive fear conditioning is studied in humans, particularly the interaction between the hippocampus and the amygdala. One important finding is that, while the amygdala appears to be sensitive to aversive stimuli generally, the hippocampus is selectively activated for *signaled* as compared to *unsignaled* aversive stimuli. That is, when an organism experiences an aversive stimulus that is paired with a sensory cue, the hippocampus is sensitive to that correlation, whereas the amygdala is active with an aversive stimulus whether it is cued or not.

Further exploration of this circuit has occurred within the phenomenon of *fear extinction*. A previous fear association can be “extinguished” when the cue previously associated with the aversive stimulus is presented without that stimulus. Even further, extinction itself can be context-sensitive; i.e., a stimulus can be unpaired from an aversive response in some contexts but not in all. Fear extinction of this context-sensitive type is interrupted by injury to the hippocampus. Moreover, injuries to the hippocampus after fear extinction inhibit *re-implementation* of the fear in non-extinguished contexts. The interaction between context recognition in the hippocampus and its “gating” of cued associations in the amygdala is posited to be impaired in contextual fear in individuals with PTSD.

Another example comes from lesion studies in mice. Wu et al. (2020) studied a delayed-match-to-sample task in which a mouse must remember a stimulus during a delay period, and then compare it to a second stimulus. One behavior, in this case a lick to a left target, is rewarded if the stimuli match, and another (lick to a right target) is if they do not. This task setting implements a kind of context sensitivity in the association between the second stimulus and the action. Whether the second stimulus needs to be responded to with a left or a right lick depends on the identity of the first stimulus. So, motor areas involved in licking responses must modulate their association between stimulus and response depending on the context.

These kinds of context-dependent behaviors can be used to show where particular aspects of a decision are implemented, and how they specifically are interrupted by injury. For instance, one possibility is that the match or non-match decision is “made” in frontal cortical areas, and then propagated to motor areas such as the ALM, which simply implements the association between second cue and appropriate response. Another possibility is that the ALM itself is involved in computing whether the stimuli match or not, only receiving information about the identity of the first stimulus from other areas.

These alternatives were tested by varying where precise, pharmacologically induced lesions were introduced during specific trials. For instance, the first possibility mentioned above suggests that lesions to the ALM during stimulus presentation or delay should *not* affect behavior, because the ALM is only relevant after the decision has been made, whereas lesions to frontal areas during the delay would impair performance. But this is the *opposite* of what was found. Lesions to the ALM during stimulus onset and the delay impaired behavior, proportional to the duration of the induced lesion. Conversely, lesions to frontal areas such as the orbitofrontal cortex only affected behavior during onset of the initial stimulus, not during the delay. Further, lesions to the ALM did *not* impair simple associations between stimulus and response, i.e., ones that were not part of a delayed match to sample task.

In each of these two examples we see the difference between a context-recognition element in the system and either an input or an output to that system. In the memory gating system, the hippocampus recognizes the context of a signaled association, or whether an association has now been subject to extinction, and gates the memory in the amygdala accordingly. In the frontal-ALM circuit, the researchers instead discovered that the frontal areas only recognized the inputs and relayed them to ALM, which in turn implemented the context-sensitive decision. In each case, the relevant systems are functionally decomposed.

Of course, each of these systems is only acting within a broader network of brain areas, so we now turn to discuss how such broader networks might be decomposed.

### Context-specific network reconfiguration

Senden et al. (2018) performed a network analysis of functional connectivity in cortical areas, specifically with regards to how specific tasks are implemented. Functional connectivity is a measure of the temporal co-activation of brain areas. Each individual area is a node, and when two areas exhibit functional connectivity, this constitutes an edge. This allows for network-theoretic measurements to be applied to neural activity as opposed to bare structural connection, and hence track, as the authors suggest, informational exchange between areas. Importantly, these networks show general topological features of the types we have discussed. For instance, a set of areas, overlapping with but not coextensive with the brain’s “default mode” network, comprises a rich club – recall, this is the kind of network where hub nodes are themselves richly interconnected.

In particular, the researchers studied *changes* in context, including the change between rest and task conditions, and a comparison of the different task conditions. They found an intriguing set of results. Analyzing the temporal sequence of activation – the pattern of how functional connectivity changes over time, can give a sense of the directionality of activity. The predominant directionality of activity in the network changed between rest and task. While the rich club received similar levels of input across conditions, it exerted much more influence on non-rich club “peripheral” nodes in task conditions. Further, while there was a significant (but not complete) overlap between the areas activated across the different types of task, the interactions *between* those areas varied depending on the task.

Here is an interpretation of these results, in line with that given by the researchers. The rich club serves as a contextual control system. When a particular task, with its particular informational requirements, is being performed, the out-degree of the rich club increases, enforcing a type of functional interaction between the components. These areas then respond in appropriate ways for that type of context. While this explanation is of course sketchy, we can see here both a distinction between control-and task-specific subsystems, with directional interactions between them, organized for the purposes of a specific task.

Slightly more detail can be seen in a study of episodic memory by Watrous et al. (Schedlbauer et al., 2014). They had subjects “navigate” around a virtual environment, dropping off and picking up a virtual friend at a series of stores. Then subjects’ functional connectivity networks were measured while they answered distinct questions about their experience. Some of the questions were *spatial* – e.g., which store was closest to store x? Some were *temporal* – e.g., which store did you visit after store x? While a broad network was activated in each context, some key points distinguished the two. First, while the medial temporal lobe, comprising the hippocampus and associated cortical regions, was an equally significant hub in the functional connectivity networks in both kinds of tasks, different areas – the lateral prefrontal cortex and the posterior parietal cortex, achieved greater network centrality in the temporal and spatial contexts, respectively.^3^ Again, the interpretation is that the medial temporal lobe serves as a context-reinstating device, organizing the network so as to recall the particular kinds of information needed for the task. Hence, one way that broad networks can be decomposed is in situations of context-sensitivity is to look for the parts of the network that mediate the context, and those that implement task-specific organization.

### Dynamic regime shift

The results in the last subsection were discussed at the broad network level, but there is also significant evidence that individual areas vary their behavior to implement the right informational requirements for specific contexts. In addition to the re-mapping results discussed in section 4.1, populations of cells can also change their *dynamical regimes* to represent information in the way required for the context.

To take one example, Warden and Miller (2010) studied working memory in monkeys’ prefrontal cortical cells. They had two tasks, both of which involved an initial presentation of a sequence of two objects. In the “recognition” task, a delay would be followed by presentation of a second sequence of objects, and the monkey would have to indicate whether the second sequence matched the first. In the “recall” task, after the delay the monkeys were presented with a set of objects and would have to re-create the sequence by making saccades to the two formerly presented objects in the right order. Object-selective cells in the prefrontal cortex behaved differently in these two contexts, specifically in the delay between the presentation of the original sequence and the presentation of the test stimulus.

In the recognition task, activity amongst the cells selective for the second object during the delay was much greater than that of the cells selective for the first object. In the recall task, however, this selectivity was equal. Why would this be? The authors suggest that in the former task, the cells operate with a “passive buffer” type of memory, where the activity of object-selective cells decays over time. This more passive type of memory suffices for the task because there is only one subsequent test stimulus that either matches that selectivity or does not. The recall task, however, requires a more active form of maintenance, since the match must be selected by the monkey out of a number of presented stimuli.

Intriguingly, this change in the dynamics of the representation – from passively decaying to actively maintained – seems to depend on cells dedicated to context-recognition. Particular cells actively represent the task context, and in turn influence the object-selective cells. Here, again, this time within a cell population, we have a part of the system that is recognizing the context, and using it to implement a specific functional change, in this case the passive versus active maintenance of information. Meyers et al. (2012) have in turn shown how task-selective cells develop in the population over the course of task-learning.

This basic idea of changing dynamic regimes has been generalized in theoretical work. Rigotti et al. (2010) modeled a neural population as comprising two sub-networks, a *context network* and an *associative network*. The associative network would learn and implement simple associations between conditioned and unconditioned stimuli. The context network, on the other hand, comprised a fully interconnected group of cells with mixed selectivity for both external events (presentation of stimuli and reward) and the states of the associative network. This allowed the context network to track what combinations of external cues and associative network states led to reward. By providing feedback to the associative network, the context network cells were able to create groupings of associations that were specific to each context. The authors show how these properties capture the kind of physiology observed in prefrontal cortical populations in a reverse-conditioning task, where original learned associations between cues and rewards are flipped in a subsequent learning epoch.

Importantly, this process affects the dynamics in the system, through a process of what the authors refer to as “attractor concretion.” This is the way in which reverbatory activity in the network will be qualitatively distinct in one context rather than another, thus implementing a distinct pattern of activity for each context. In this way, a distributed network with distinct populations can implement context-specific dynamics.

## Argument from the cases

I have argued, first, that the question of whether context sensitivity is incompatible with reductive/mechanistic explanation turns on whether we can discover mechanisms that implement new forms of organization for specific contexts. Second, I have argued that there are many types of such mechanisms. The question is how to interpret these cases.

My phrasing of reductionism, espoused in section 2, is that of *pragmatic downward pull*. This is the normative principle that a full understanding of a system requires decomposing the system at lower levels. The cases above suggest that it is possible to do this. In each case, there are specific cells, populations, or sub-networks that implement the contextual changes in the network, and components that shift their function in response to those changes. Of course, these decompositions are not simple, easy, or atomistic. The process of doing functional decomposition in these systems is much more complicated than in the toy *Plinko* case I gave in section 3. But, on the pragmatic view of the debate, complexity does not just equal emergence. It is a claim about what the most successful science does.

Nor have I suggested that any of these explanations are *complete*. There are many more details to fill in, many new contexts to understand, etc. In particular, the way that contextual changes result in particular functional patterns is better understood at the circuit level than at the network level – what, for instance, is it about the new functional organizations of peripheral nodes in the Senden et al. study that enables the specific tasks in which they are implemented? The reductionist picture’s *pragmatic downward* aspect suggests that further study of these contexts should proceed until the kinds of explanations that have been given at the cell population level are possible.

The emergentist is forced into an awkward situation with regards to these mechanisms. They must either deny that they really are mechanisms, or they must say that we do not really learn anything from discovering them. I do not think either option is tenable. If we agree that decomposition and localization are definitive of mechanistic/reductionistic explanation, then these analyses which assign particular functions to distinct cells, populations, or subnetworks are mechanistic, despite the fact that these decompositions do not posit immutable, fixed mechanisms, but rather contextually shifting ones. It would be hard to know how to adjudicate the claim that we do not learn anything important from these analyses. Remember, the pragmatic argument is an abduction from successful scientific practices. These practices, if successful in the mechanistic sense, can only be ruled out of bounds by assuming that mechanistic analysis is not productive, which is just what is under consideration.

There are a couple of strategies left open to the emergentist at this point, which I’ll call the *shifting domain* strategy, and the *shifting explanandum* strategy. Frequently emergentists make nods to mechanistic analysis – *sometimes*, they admit (e.g., for simple interactions within the system), mechanistic explanation is possible, useful, etc. But they then suggest that for the circumstances that are really interesting for understanding biological function, these approaches must be left aside. So, is there any principled way of defining a series of settings where mechanistic/reductive explanation is not useful, even if it is in explaining the kind of behavioral phenomena I discussed above?

The shifting domain strategy suggests that for certain kinds of phenomena, mechanistic/reductive explanation is bound to fail, even if it is successful in other cases. Psychopathology is one such domain often referred to by Silberstein et al – here the idea is that psychiatry is the kind of domain for which the mechanistic facts about the system drop out of the explanation, and all of the explanatory work is to be done by contextual and topological/dynamical properties. So can such a move help parcel explanations into those that are amenable to mechanism and those that aren’t?

There is no doubt that some significant advances in studying psychopathology at the neural level have been achieved by employing network frameworks, including studies of the rich club and the way it is interrupted in such cases as schizophrenia (van den Heuvel et al., 2013). But, given the current state of the dialectic, this bare fact is far from sufficient to establish the emergentist conclusion. Often, the way the argument goes in these cases is that the emergentist contrasts the topological approach with the kind of reductive explanation that seeks, for instance, a single genetic or neural locus for psychiatric disease. If a “biopsychosocial” model is right, they contend, then mechanistic explanation is impossible.

On reflection however, this argument illicitly assumes that that traditional atomistic model of reduction is the only one possible. Nothing about the more sophisticated forms of reductionist/mechanistic explanation denies that topological description can play *a* role, even a critical role, in explaining the phenomenon. Nor does it ally itself with single-locus analyses of pathology, or deny that social/developmental factors may vitally influence the mechanisms responsible for it. The pragmatic downward pull approach suggests that our explanations will be deeper and better if we seek lower-level explanations in addition to the higher-level ones.

We already saw above how the rich club has been implicated in the way that distinct tasks are mediated by reorganizations of peripheral nodes. A natural hypothesis is that interruption of the rich club network in schizophrenia affects these reorganizations. But in order to understand this, we’d need to *both* understand how informational reorganizations operate in the regular tasks, and how that normal operation is interrupted in schizophrenia. The pragmatic downward pull approach, in my view correctly, normatively suggests searching for those explanations, and that decomposition and localization (of the contextualized sort) are reasonable explanatory strategies for pursuing them. The emergentist approach rules this out by fiat.

The explanandum shifting strategy suggests that there are certain properties *of the system* that are only explicable by (for example) network frameworks, and not by mechanistic frameworks. A set of properties that has frequently been adduced in this setting includes the system being *robust*, the system having a certain kind of dynamics, or the system exhibiting scale dependence in how it is modeled (Green and Batterman, 2017). The idea here is that when we look at properties of a class of systems themselves, rather than the phenomena they produce, we will have to resort to network and dynamical descriptions at the expense of mechanistic ones. Again, no one should doubt the importance of network description in these contexts. But again, we can question whether this has the overall upshot that the emergentist assumes.

I suggest that this way of arguing implicitly assumes a version of explanatory pluralism that is contestable, and hence the argument does not go through without a prior establishment of a thesis about pluralism, which regularly goes undiscussed in these contexts. On what I’ll call “division-of-labor” pluralism (Potochnik, 2017; Rathkopf, 2018; Burnston, 2019), there are distinct explananda we might investigate about a system, and those distinct explananda will require distinct and disjoint types of explanation. When one changes explananda – for instance, in switching from an explanation of how memory occurs, to asking how memories can be robust to patterns of decay and variation –one selects the right kind of explanatory framework for that explanandum. But the division-of-labor view is not the only version of pluralism.

On more “integrative” approaches, distinct kinds of explanations or models need to contribute even to understanding one single explanandum. If one embraces an integrative view, then understanding the relationship between mechanistic description and network description of the system is required. On this kind of view, a system property like robustness will be better explained by taking into account both network features and the particular, functionally distinct roles played by their constituents. So, explaining how a memory can be retained in some contexts while extinguished in others, or how a monkey learns to recognize a type of maze despite superficial variations, can only be achieved by both analyzing the brain networks involved *and* the causal/functional specifics of the constituents of the network. In any event, the division-of-labor view cannot just be presumed to be more fruitful than more integrative views, and hence the adjudication of the explanandum-shifting strategy has to be pursued within the larger discussion of scientific pluralism.

## Conclusion

Recent projects have argued from the presence of complexity in biological systems to the presence of emergence, and the concomitant failure of mechanistic decomposition, in these systems. I have argued that this argumentative move is by no means obvious, particularly if we focus on the mechanisms involved in contextual reorganization of these systems. If I am right, then there is no easy argument from context-sensitivity to emergence.

## Data availability statement

The original contributions presented in the study are included in the article/Supplementary material, further inquiries can be directed to the corresponding author.

## Author contributions

The author confirms being the sole contributor of this work and has approved it for publication.

## Conflict of interest

The author declares that the research was conducted in the absence of any commercial or financial relationships that could be construed as a potential conflict of interest.

## Publisher’s note

All claims expressed in this article are solely those of the authors and do not necessarily represent those of their affiliated organizations, or those of the publisher, the editors and the reviewers. Any product that may be evaluated in this article, or claim that may be made by its manufacturer, is not guaranteed or endorsed by the publisher.

## Footnotes

^1^While the distinction between explanatory and "theoretical" reduction has become entrenched, I do not actually think it is all that deep. Mechanistic models, on my view, are parts of theories about how the system is organized. I will not pursue this further here, though.

^2^We can of course imagine more complicated, or *themselves varying*, forms of interaction, but the question is, similarly, here, whether these contextual shifts are mechanically mediated. Suppose, in addition to the lever, there is a switch. When the switch is thrown the obstacles exert a slight magnetic attraction on the ball. This will of course change the kinds of paths the ball exhibits. But the switch explains the introduction of the magnetic attraction, and the new paths will be determined by the new type of interaction between the ball and the obstacles. There is nothing here that is not mechanistically explicable.

^3^This was accompanied by a distinctive change in the background local field potential at which the network synchronized, further dissociating the contexts. See Burnston, 2021, for more details.
